# βig-h3 Promotes Human Osteosarcoma Cells Metastasis by Interacting with Integrin α2β1 and Activating PI3K Signaling Pathway

**DOI:** 10.1371/journal.pone.0090220

**Published:** 2014-03-04

**Authors:** Yun-Shan Guo, Rui Zhao, Jie Ma, Wei Cui, Zhen Sun, Bo Gao, Shu He, Yue-Hu Han, Jing Fan, Liu Yang, Juan Tang, Zhuo-Jing Luo

**Affiliations:** 1 Department of Osteology, Xijing Hospital, Fourth Military Medical University, Xi'an, China; 2 Cell Engineering Research Centre & Department of Cell Biology, State Key Laboratory of Cancer Biology, State Key Discipline of Cell Biology, Fourth Military Medical University, Xi'an, China; 3 Department of Neurosurgery, Tangdu Hospital, Fourth Military Medical University, Xi'an, China; 4 Department of Endocrinology and Metabolism, Xijing Hospital, Fourth Military Medical University, Xi'an, China; Aix-Marseille University, France

## Abstract

Osteosarcoma, the most common primary bone tumor in children and young adolescents, is characterized by local invasion and distant metastasis. But the detailed mechanisms of osteosarcoma metastasis are not well known. In the present study, we found that βig-h3 promotes metastatic potential of human osteosarcoma cells in vitro and in vivo. Furthermore, βig-h3 co-localized with integrin α2β1 in osteosarcoma cells. But βig-h3 did not change integrin α2β1 expression in Saos-2 cells. Interaction of βig-h3 with integrin α2β1 mediates metastasis of human osteosarcoma cells. The second FAS1 domain of βig-h3 but not the first FAS1 domain, the third FAS1 domain or the fourth FAS1 domain mediates human osteosarcoma cells metastasis, which is the α2β1 integrin-interacting domain. We further demonstrated that PI3K/AKT signaling pathway is involved in βig-h3-induced human osteosarcoma cells metastasis process. Together, these results reveal βig-h3 enhances the metastasis potentials of human osteosarcoma cells via integrin α2β1-mediated PI3K/AKT signal pathways. The discovery of βig-h3-mediated pathway helps us to understand the mechanism of human osteosarcoma metastasis and provides evidence for the possibility that βig-h3 can be a potential therapeutic target for osteosarcoma treatment.

## Introduction

Osteosarcoma is a high-grade malignant bone neoplasm that occurs primarily in children and young adolescents. It occurs with an incidence of approximately three cases per million people per year [1]. The principles of treatment of osteosarcoma have undergone dramatic improves in the past 20 years. Multi-agent chemotherapy increased the 5-year overall survival of patients with localized disease to between 60% and 78% [2]. The survival of patients with metastatic osteosarcoma, however, remains poor with survival rates ranging from 11% to 20% [3], [4]. This outcome suggested that 80% of the patients had metastasis at the time of presentation. Hence, a novel strategy that would efficiently inhibit osteosarcoma metastasis is highly desirable.

Tumor metastasis consists of a trail of complex procedures, all of which must be successfully completed to result in clinically detectable metastatic tumors at distal tissues [5], [6]. To complete the process, primary cancer cells have to attach to extracellular matrix (ECM) components, invade through the basement membrane, intravasate into the circulation, and extravasate to distal tissues [7], [8]. The entire process regulated by interactions between cancer cells and ECM. As a major component of the tumor microenvironment, ECM proteins potentially affect the metastasis process [9]. Thus, molecular alterations of the ECM proteins in the tumor microenvironment have a considerable impact on the metastatic process during tumorigenesis.

Transforming growth factor (TGF)-β-inducible gene-h3 (βig-h3), which also called TGFBI, RGD-CAP, and MP78/70, is widely expressed in various types of tumor cells [10]–[12]. The βig-h3 protein was initially identified by differential screening of a cDNA library produced from A549 human lung adenocarcinoma cells treated with TGF-β [13]. The protein consists of 683 amino acids, four fasciclin-1 (FAS1) homologous domains and an RGD motif at the C-terminus [14]. The FAS1 domains are homologous to fasciclin-1 in Drosophila and well conserved in several proteins from different species. FAS1 domain motif containing proteins, including βig-h3, participate in cellular function via interactions with various integrins, including integrin a3β1, integrin αvβ3, and integrin avβ5 [15]–[17]. As an ECM protein, βig-h3 is involved in cell proliferation, migration, apoptosis and differentiation, and might function as either a promoter or an inhibitor of carcinogenesis, depending on cells and tumor types [18]–[21]. The gain or loss of expression of βig-h3 might be involved in tumor formation and acquisition of a metastatic phenotype in human cancer. Although, previous studies have reported that βig-h3 is required for apoptosis in human osteosarcoma cells [22], it is not clear yet whether βig-h3 is involved in osteosarcoma metastasis.

This study sought to examine whether βig-h3 expression could influence osteosarcoma cells metastasis and to determine the molecular mechanism by which this occurred, in an effort to elucidate the role of βig-h3 in the regulation of osteosarcoma metastasis. In the present study, we showed that βig-h3 promotes adhesion, invasion and migration of human osteosarcoma cells. βig-h3 mediates human osteosarcoma cells metastasis through interacting with integrin α2β1, and then activates downstream PI3K/AKT signaling pathway. Furthermore, we identified that only the second FAS1domain of βig-h3 was involved in osteosarcoma cells metastasis.

## Results

### Downregulation of βig-h3 decreases adhesion, invasion and migration of human osteosarcoma cells in vitro

**Figure 1:**
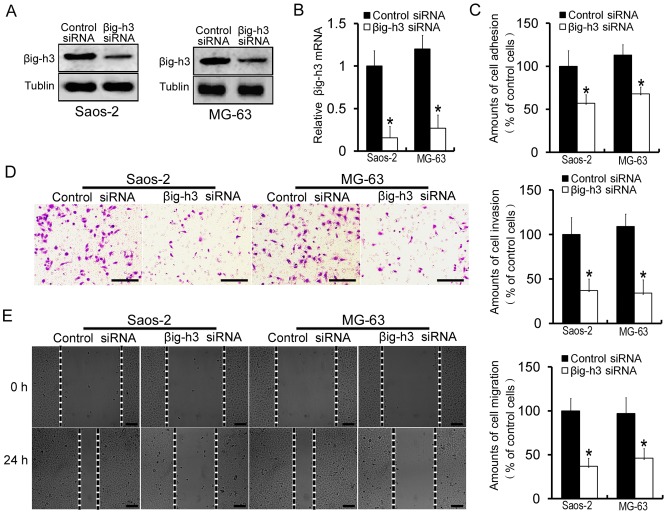
Downregulation of βig-h3 decreases adhesion, invasion and migration of human osteosarcoma cells in vitro. (A) Western blot was performed to examine the βig-h3 protein levels in Saos-2 cells and MG63 cells which were transfected with control siRNA or βig-h3 siRNA. (B) Real Time PCR was performed to examine the βig-h3 mRNA levels in Saos-2 cells and MG63 cells which were transfected with control siRNA or βig-h3 siRNA. (C) The amounts of cell adhesion was tested in Saos-2 cells and MG63 cells which were transfected with control siRNA or βig-h3 siRNA. (D) The amounts of cell invasion was tested in Saos-2 cells and MG63 cells which were transfected with control siRNA or βig-h3 siRNA. (E) The amounts of cell migration was tested in Saos-2 cells and MG63 cells which were transfected with control siRNA or βig-h3 siRNA. Control siRNA were used as a negative control. Scale = 100 µm. The adhension assay, invasion assay, and migration assay were adopted as described in Materials and Methods. Values are the means±SE from six independent experiments. *P<0.05 by Student's t test.

As an ECM protein, βig-h3 is involved in cell proliferation, migration, invasion, apoptosis and tumorigenesis [18]–[21]. To test the role of βig-h3 in human osteosarcoma cells, small interfering RNAs against βig-h3 (βig-h3 siRNA) were transfected into the human osteosarcoma cell lines, Saos-2 cells and MG63 cells, for 48 hours to knockdown βig-h3 mRNA and protein expression. Silencer negative control siRNAs (control siRNA) were also used as a negative control. As compared with control siRNA treated cells, the βig-h3 siRNA could effectively decrease the mRNA and protein expression of βig-h3 in Saos-2 cells and MG63 cells (P<0.05, Figure 1A and 1B). Cell function assays demonstrated that the amounts of cell adhesion were significantly decreased after βig-h3 siRNA treatment in Saos-2 cells and MG63 cells (57.6%±11.9% and 52.3%±9.4%, respectively) (P<0.05, Figure 1C). In addition, the abilities of cells to invade through Transwell chambers were decreased after transfected with βig-h3 siRNA in Saos-2 cells and MG63 cells. (37.1%±18.5% and 31.2%±12.8%, respectively) (P<0.05, Figure 1D). Similarly, treatment with βig-h3 siRNA also decreased the amounts of cell migration in Saos-2 cells and MG63 cells (39.2%±15.3% and 45.4%±10.7%, respectively) (P<0.05, Figure 1E). This finding suggests that βig-h3 may enhance adhesion, invasion and migration potential of human osteosarcoma cells.

### βig-h3 promotes metastasis of human osteosarcoma cells in vivo

**Figure 2:**
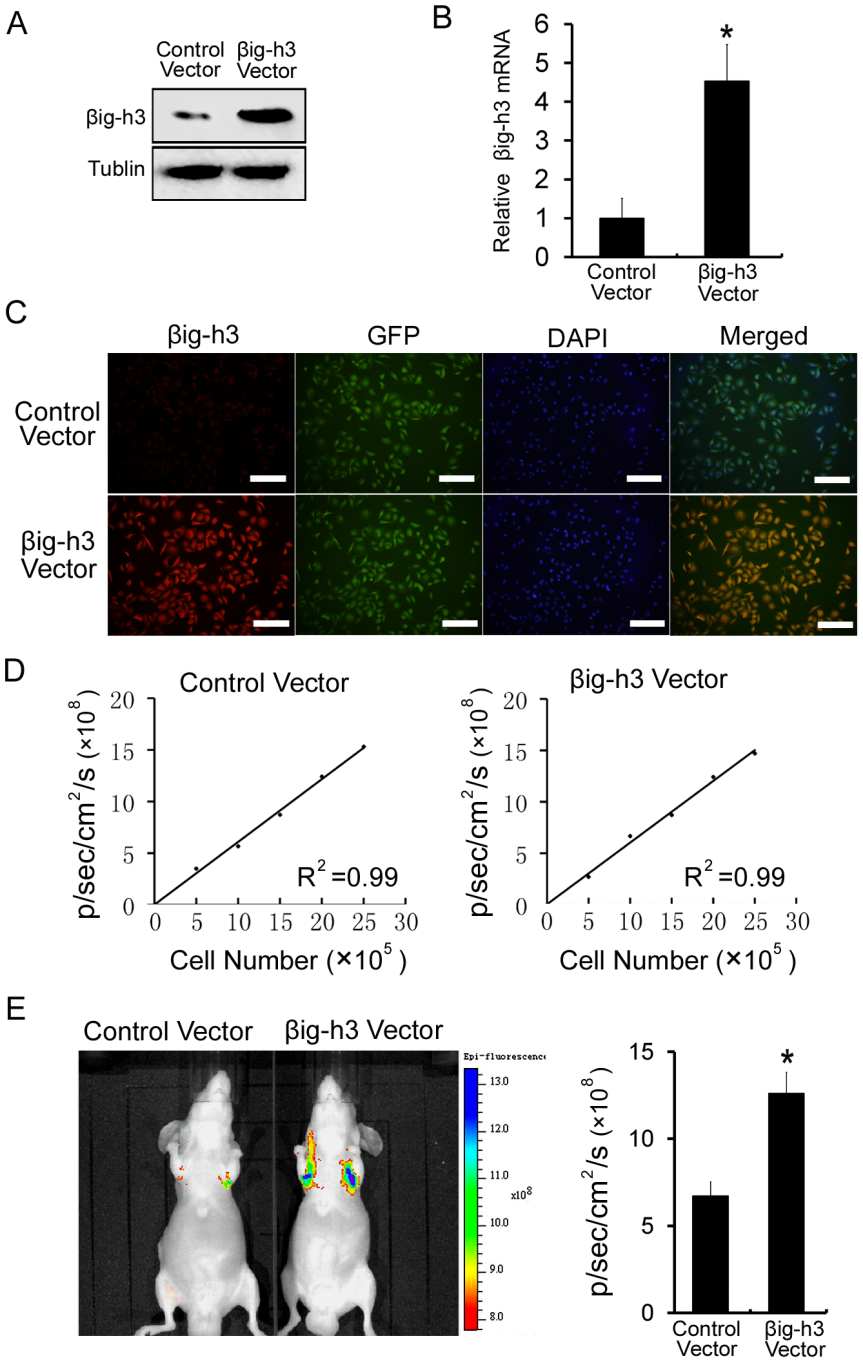
βig-h3 promotes metastasis of human osteosarcoma cells in vivo. (A) Western blot was performed to examine the βig-h3 protein levels in Saos-2 cells which were transfected with control vector or βig-h3 vector. (B) Real Time PCR was performed to examine the βig-h3 mRNA levels in Saos-2 cells which were transfected with control vector or βig-h3 vector. (C) Immunofluorescence was performed to examine the expression levels of GFP and βig-h3 in Saos-2 cells which were transfected with control vector or βig-h3 vector. Scale = 100 µm. (D) A strong correlation exists between the cell number and GFP fluorescence intensity (control vector treated cells, R2 = 0.99; βig-h3 vector treated cells, R2 = 0.99). (E) Representative fluorescence imaging of mice injected with Saos-2 cells stably expressing control vector or βig-h3 vector. Quantification of fluorescence imaging data is shown at the right. Bars represent the mean of triplicate samples; error bars represent standard deviation. Data are representative of three independent experiments. *P<0.05 by Student's t test.

To extend these studies in vivo, Saos-2 cells stably expressing GFP were established by lentiviral infection. Then the βig-h3 vector and a control vector were stably transfected into Saos-2 cells which were stably expressing GFP. As compared with control vector-treated cells, the βig-h3 vector could stably increase the mRNA and protein expression of βig-h3 in Saos-2 cells (P<0.05, Figure 2A and 2B). Moreover, we observed stable expression of GFP and a strong correlation between GFP fluorescence signals and cell number in both control vector-treated cells and βig-h3 vector-treated cells (Figure 2C and Figure 2D). Furthermore, Saos-2 cells expressing the GFP were injected into immunodeficient mice through the tail vein. GFP fluorescence imaging was used to monitor the presence of Saos-2 cells. Due to size restrictions imposed by mouse capillaries, human tumor cells are rarely able to pass from the venous to the arterial system by way of the lung. Cells that failed to metastasize were not able to survive. Detectable GFP fluorescence signals indicated that cells had succeeded in metastasizing [23]–[24]. We found that the GFP signal in the group of βig-h3 vector-transfected cells was significantly higher than the GFP signal in the group of control vector-transfected cells in the lung (P<0.05, Figure 2E). Therefore, the result indicated that βig-h3 significantly promotes metastasis of human osteosarcoma cells in vivo.

### βig-h3 immunoprecipitates with α2β1 integrin in human osteosarcoma cells

**Figure 3:**
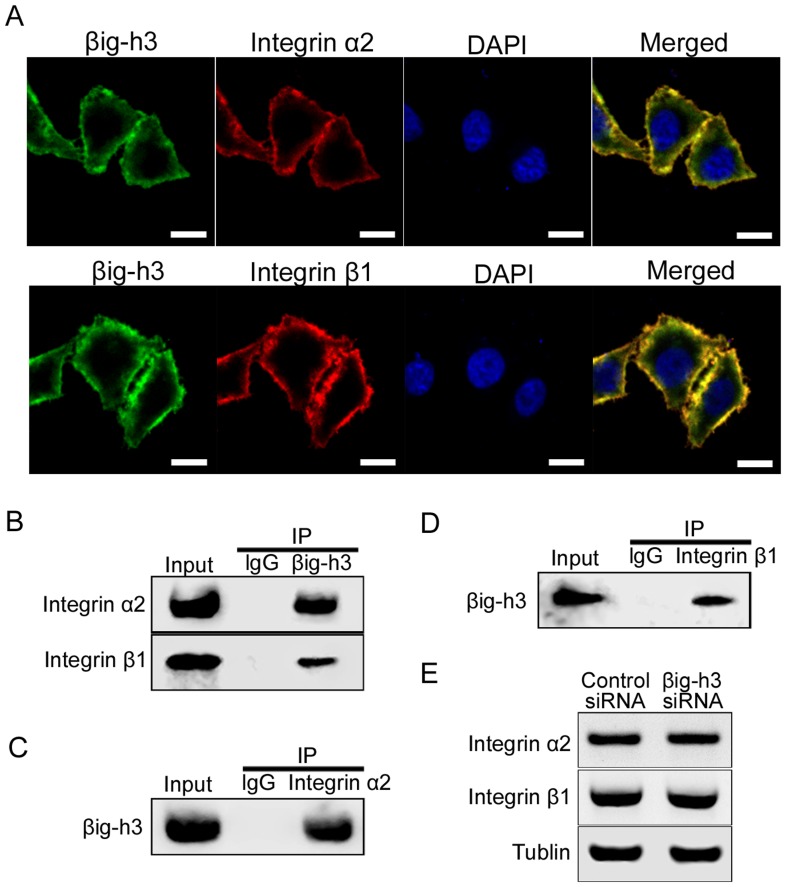
βig-h3 immunoprecipitates with α2β1 integrin in human osteosarcoma cells. (A) Localization of βig-h3 and integrin α2β1 in Saos-2 cells. Saos-2 cells were double-stained for βig-h3 (green) and integrin α2 and integrin β1 (red). Scale = 2 µm. (B) βig-h3 immunoprecipitation with integrin α2β1. Lysates of Saos-2 cells were subjected to immunoprecipitation with anti- βig-h3 antibody pre-bound coupling gel, integrin α2 and integrin β1 in the immune complexes were detected by western blot analysis. (C) Integrin α2 immunoprecipitation with βig-h3. Lysates of Saos-2 cells were subjected to immunoprecipitation with anti-integrin α2 antibody pre-bound coupling gel, βig-h3 in the immune complexes was detected by Western blot analysis. (D) Integrin β1 immunoprecipitation with βig-h3. Lysates of Saos-2 cells were subjected to immunoprecipitation with anti-integrin β1 antibody pre-bound coupling gel, βig-h3 in the immune complexes was detected by Western blot analysis. Immunoprecipitated with anti-IgG antibody was used as the negative control. (E) Western blot was performed to examine the integrin α2 and integrin β1 protein levels in Saos-2 cells which were transfected with control siRNA or βig-h3 siRNA.

Integrins are cell surface adhesive receptors composed of α and β chain heterocomplexes, which play an essential role in osteosarcoma metastasis [25]–[28]. It has been reported that both α2 and β1 subunits are expressed in human osteosarcoma cells and they serve as bidirectional transducers of extracellular and intracellular signals in tumor metastasis processes [29]–[31]. Accordingly, we hypothesized that βig-h3 might interact with integrin α2β1 to affect the metastasis ability of osteosarcoma cells. Immunofluorescent double staining was performed to examine cellular distribution of integrin α2β1 and βig-h3 in Saos-2 cells. The results showed co-localizations of βig-h3 with integrin α2 and integrin β1 subunits on the cell membrane (Figure 3A). To further confirm this result, co-immunoprecipitation assay were performed to detect the interaction of βig-h3 with integrin α2 and integrin β1 subunits. integrin α2 and integrin β1 subunits were found to co-immunoprecipitate with endogenous βig-h3 in Saos-2 cells lysates, (Figure 3B–3D), indicating that βig-h3 and integrin α2β1 interact in their native conformations. To elucidate the effects of βig-h3 on integrin α2β1 expression, we tested the protein expressions of integrin α2 and integrin β1 subunits in βig-h3 siRNA transfected Saos-2 cells. We found that there were no significant expression modifications of integrin α2 and integrin β1 in βig-h3 siRNA transfected cells compared with control cells (Figure 3E). This finding suggested that βig-h3 did not change integrin α2β1 expression in Saos-2 cells.

### Interaction of βig-h3 with integrin α2β1 mediates metastasis of human osteosarcoma cells

**Figure 4:**
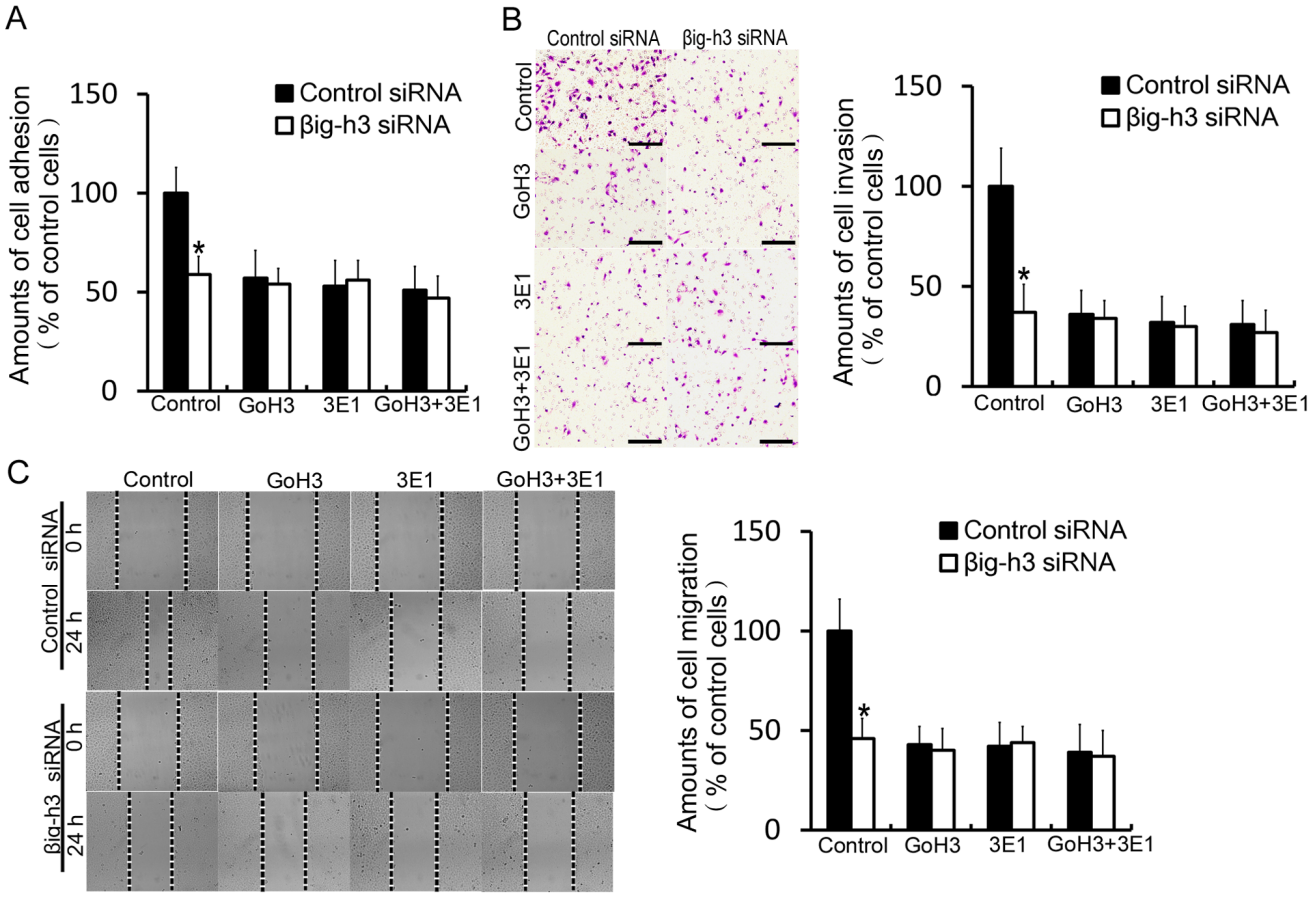
Interaction of βig-h3 with integrin α2β1 mediates metastasis of human osteosarcoma cells. The amounts of cell adhesion (A), invasion (B) and migration (C) were tested in control siRNA or βig-h3 siRNA transfected Saos-2 cells which were incubated with P1E6 and 6S6, alone or combination. Scale = 100 µm. Bars represent the mean of triplicate samples; error bars represent standard deviation. Data are representative of three independent experiments. *P<0.05 by one-way ANOVA analysis.

To identify whether integrin α2β1 is involved in βig-h3 mediated human osteosarcoma metastasis, the function blocking antibodies, mouse anti-human integrin α2 mAb (P1E6) and mouse anti-human integrin β1 mAb (6S6) were used. We found that βig-h3 siRNA markedly reduced the amounts of cell adhesion in blank control group (p<0.05, Figure 4A). However, the addition of P1E6 and 6S6, alone or combination reduced the amounts of cell adhesion in control siRNA transfected cells to levels comparable with that in βig-h3 siRNA transfected cells. βig-h3 siRNA did not further reduce the amounts of cell adhesion after blocking integrin α2β1 (p>0.05, Figure 4A). The result indicated that integrin α2β1 is involved in βig-h3 mediated cell adhesion. In addition, invasion and migration assay were performed. We found that βig-h3 siRNA markedly reduced the amounts of cell invasion and migration in blank control groups (p<0.05, Figure 4B and 4C). However, the addition of P1E6 and 6S6, alone or combination reduced the amounts of cell invasion and migration in control siRNA transfected cells to levels comparable with that in βig-h3 siRNA transfected cells. βig-h3 siRNA did not further reduce the amounts of cell invasion and migration after blocking integrin α2β1 (p>0.05, Figure 4B and 4C). The above results indicate that integrin α2β1 is required for βig-h3 mediates metastasis of human osteosarcoma cells.

### The second FAS1 domain of βig-h3 promotes human osteosarcoma cells metastasis

**Figure 5:**
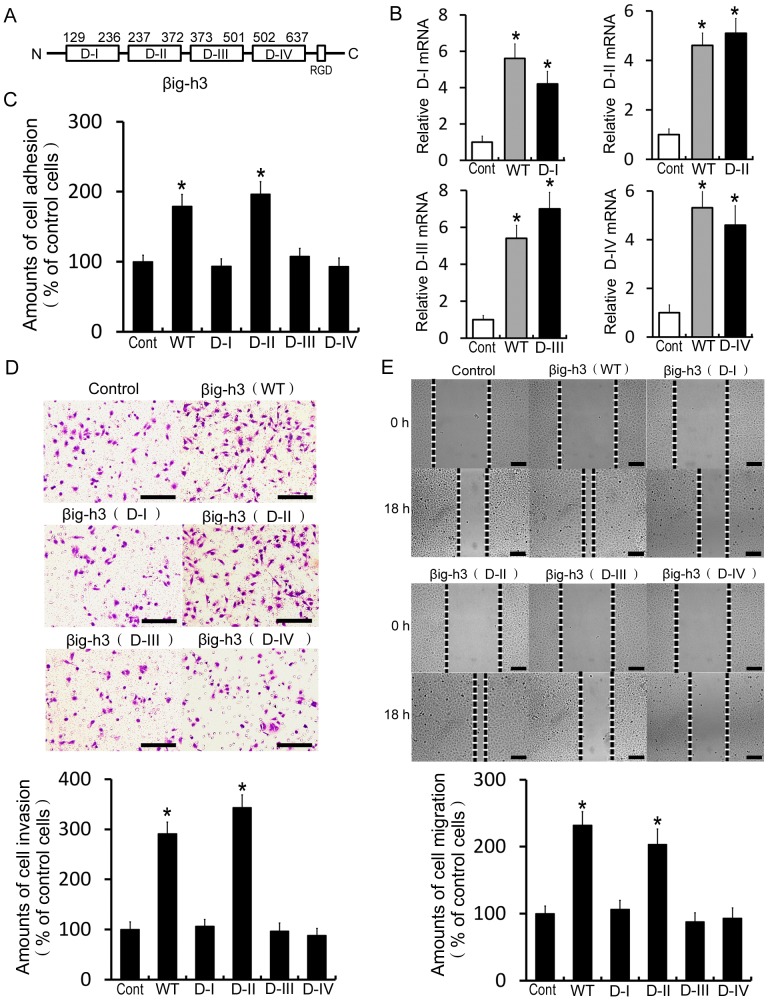
The second FAS1 domain of βig-h3 promotes human osteosarcoma cells metastasis. (A) Schematic representation of the total gene of βig-h3 (WT), the first FAS1domain (D-I),the second FAS1domain (D-II), the third FAS1domain (D-III) and the fourth FAS1domain (D-IV). (B) The total gene of βig-h3 (WT) and its four segments of highly conserved sequence (D-I, D-II, D-III and D-IV) were cloned and transfected into Saos-2 cells. Real Time PCR was used to test the mRNA expression levels of D-I, D-II, D-III and D-IV in Saos-2 cells respectively. βig-h3 (WT) transfected cells were used as positive control. The amounts of cell adhesion (C), invasion (D) and migration (E) were tested in βig-h3 (WT), D-I, D-II, D-III and D-IV transfected Saos-2 cells. Scale = 100 µm. Bars represent the mean of triplicate samples; error bars represent standard deviation. Data are representative of three independent experiments. * p<0.05 by one-way ANOVA analysis.

The βig-h3 protein consists of four FAS1 homologous domains and an RGD motif at the C-terminus [14]. The FAS1 domains are homologous to fasciclin-1 in Drosophila and participate in cellular function via interactions with various integrins [15]–[17]. To identify which FAS1domain mediated human osteosarcoma cells metastasis, we cloned the total gene of βig-h3 (WT) and its four segments of highly conserved sequence, the first FAS1domain (D-I), the second FAS1domain (D-II), the third FAS1domain (D-III) and the fourth FAS1domain (D-IV) and then we transfected them into Saos-2 cells. We found that mRNA of the four FAS1domains of βig-h3 were overexpressed in Saos-2 cells respectively (P<0.05, Figure 5B). Cell adhesion, invasion and migration assay demonstrated that overexpression of the second FAS1domain promoted the amounts of cell adhesion, invasion and migration to levels comparable with that in the total gene of βig-h3 overexpressed cells (P<0.05, Figure 5C–5E). However, overexpression of the first FAS1 domain, the third FAS1 domain and the fourth FAS1domain were not able to promote cell adhesion, invasion and migration in Saos-2 cells (P>0.05, Figure 5C–5E). These results suggested that only the second FAS1domain, but not the first FAS1domain, the third FAS1domain or the fourth FAS1domain of βig-h3 was involved in osteosarcoma cells metastasis

### βig-h3 induces human osteosarcoma cells metastasis by activating PI3K signaling pathway

**Figure 6:**
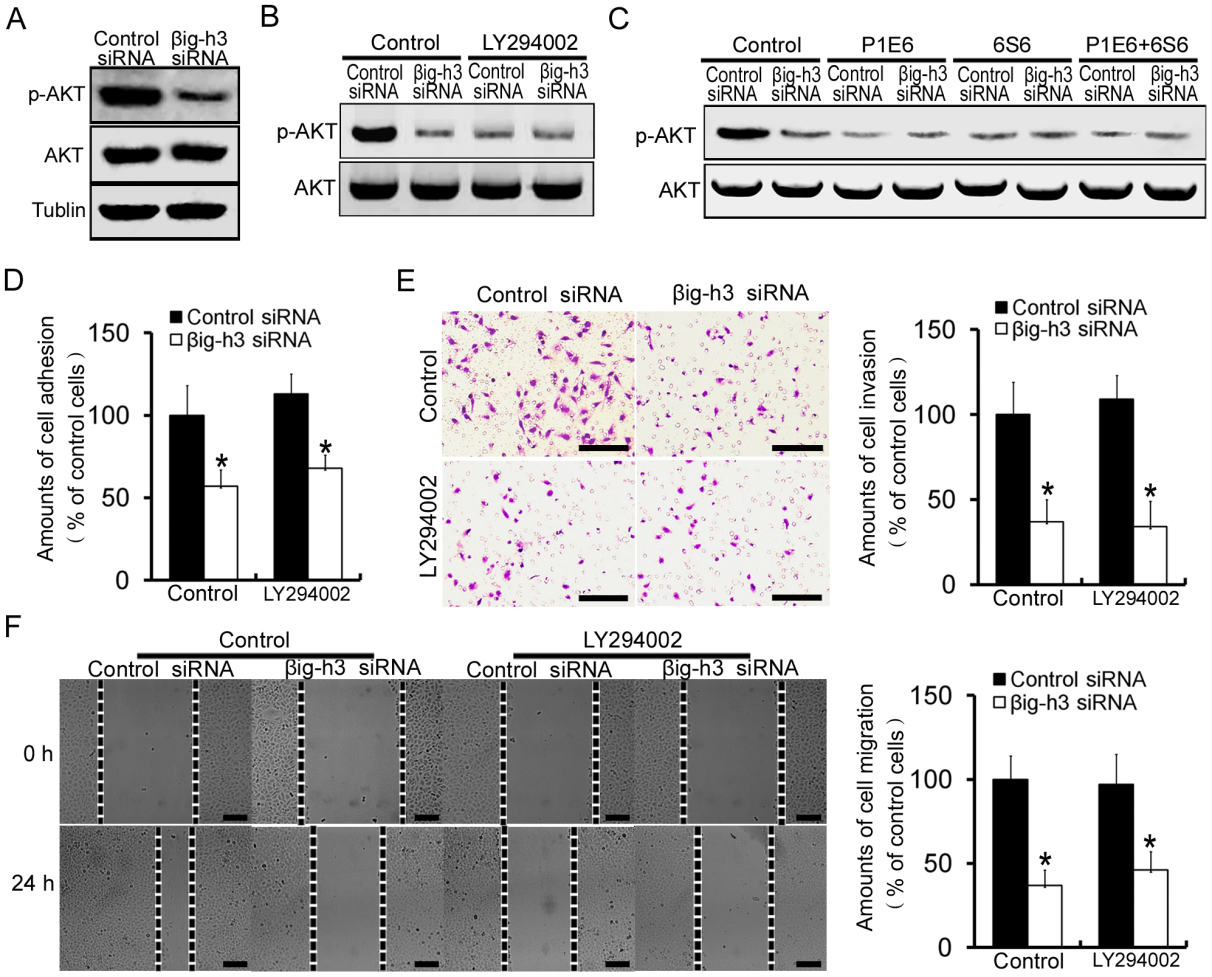
βig-h3 induces human osteosarcoma cells metastasis by activating PI3K signaling pathway. (A) Expression levels of phosphorylated AKT (p-AKT) and AKT were analyzed in control siRNA or βig-h3 siRNA transfected Saos-2 cells. (B) Expression levels p-AKT and AKT were analyzed in control siRNA or βig-h3 siRNA transfected Saos-2 cells which were incubated with PI3K inhibitor LY294002. (C) Expression levels of p-AKT and AKT were analyzed in control siRNA or βig-h3 siRNA transfected Saos-2 cells which were incubated with P1E6 and 6S6, alone or combination. The amounts of cell adhesion (D), invasion (E) and migration (F) were tested in control siRNA or βig-h3 siRNA transfected Saos-2 cells which were incubated with LY294002. Scale = 100 µm. Bars represent the mean of triplicate samples; error bars represent standard deviation. Data are representative of three independent experiments. *P<0.05 by Student's t test.

Currently, the identities of integrin α2β1-associated signaling molecules that are responsible for mediating human osteosarcoma cells metastasis in response to βig-h3 are unclear. To determine the signaling pathways that contribute to human osteosarcoma cells metastasis induced by βig-h3, an examination was conducted into the effects of βig-h3 on the phosphorylation status of AKT. Knockdown of βig-h3 was found to decrease phosphorylation of AKT in Saos-2 cells (Figure 6A). To further test whether PI3K is involved in βig-h3 mediated Saos-2 cells metastasis, LY294002, a reversible inhibitor of PI3K was employed. βig-h3 siRNA markedly reduced phosphorylation of AKT in control group. However, βig-h3 siRNA did not further reduce phosphorylation of AKT following LY294002 treatment (Figure 6B). The result suggested that activity of PI3K is required for βig-h3 induced phosphorylation of AKT. Moreover, the addition of P1E6 and 6S6, alone or combination reduced the levels of phosphorylation of AKT in control siRNA transfected cells to levels comparable with that in βig-h3 siRNA transfected cells (Figure 6C). The result indicated that integrin α2β1 is involved in βig-h3 induced phosphorylation of AKT. In addition, adhesion, invasion and migration assay were performed. We found that βig-h3 siRNA markedly reduced the amounts of cell adhesion, invasion and migration of Saos-2 cells in control groups (p<0.05, Figure 6D–6F). However, the addition of LY294002 reduced the amounts of cell adhesion, invasion and migration in control siRNA transfected cells to levels comparable with that in βig-h3 siRNA transfected cells. βig-h3 siRNA did not further reduce the amounts of cell adhesion, invasion and migration following LY294002 treatment (p>0.05, Figure 6D–6F). The above results indicated that PI3K/AKT signaling pathway is involved in βig-h3 induced human osteosarcoma cells metastasis.

## Discussion

Osteosarcoma is the most common primary bone tumor in children and young adolescents. Although significant improvements in the treatment of patients with osteosarcoma recently, patients with metastatic osteosarcoma still have very poor prognosis [1], [2]. Compounding the problem is that the molecular basis underlying metastatic osteosarcoma is poorly understood. Progression of osteosarcoma is thought to owing to cells attaching to ECM, invading through the basement membrane and migrating to distant tissues [3], [4]. Thus, selectively blocking these metastatic abilities, through targeted therapy of key molecules should be an attractive strategy to inhibit osteosarcoma metastasis.

βig-h3, an ECM protein mainly induced by TGF-β, was first identified in the human lung adenocarcinoma cell line A549 [13]. It is expressed in many tumor cells and tissues including the liver, lung, prostate and kidney [10]–[12]. Although its roles are largely unknown, it has been suggested that it is involved in the regulation of many aspects of tumor cell processes, including cell adhesion, spreading, invasion, proliferation and apoptosis [18]–[21]. In the present study, the effects of βig-h3 on cell adhesion, invasion and migration were determined in osteosarcoma cells. The cell adhesion assay revealed that knockdown of βig-h3 counteracted the adhesion of osteosarcoma cells to matrigel. Moreover, knockdown of βig-h3 effectively inhibited the cell invasion and migration of osteosarcoma cells using transwell chamber and wound healing assay. We further discovered that βig-h3 significantly promoted metastasis of human osteosarcoma cells in vivo using lung metastasis experiment. These results indicated that βig-h3 acts as a major contributor to metastatic potential of osteosarcoma.

It has been suggested that biochemical signals of βig-h3 can be transmitted across the plasma membrane through integrins to regulate various cellular functions, including adhesion, invasion, migration, survival, growth and differentiation [15]–[17]. Integrins, a large family of cell matrix adhesion receptors, have been demonstrated to play important roles in many types of tumor cells. Through the interaction with the basement membrane, integrins can mediate cell adhesion and invasion [25]–[28]. The overexpression of integrin α2β1 has been reported to be associated with poor overall survival in patients with osteosarcoma [29]–[31]. In this study, βig-h3 was found to colocalize and co-immunoprecipitate with integrin α2β1 in osteosarcoma cells. These results demonstrate that βig-h3 and integrin α2β1 at least are in proximity, if not directly associated in osteosarcoma cells. Even though the interaction of βig-h3 and integrins has been largely described in many other cellular lines [15]–[17], the exact mechanisms that link βig-h3 to integrin α2β1 have not been reported yet. Our results further show that blocking the functions of integrin α2β1 with antibodies specific for integrin α2 and β1 reduces cell adhesion, invasion and migration in control siRNA transfected cells. However, no significant inhibitory effect is obtained in βig-h3 siRNA transfected cells. These results indicate that the enhancing effect of βig-h3 on cell metastasis potential is mediated through integrin α2β1. We also demonstrated that the expression levels of integrin α2 and integrin β1 are not influenced by the expression levels of βig-h3 in osteosarcoma cells. That means the enhancing effect of βig-h3 is not mediated through the overexpression of integrin α2β1. It is known that cells can change the conformation of their integrins in response to cellular stimulation in a process often termed “integrin activation”. This conformational change mediates events such as cell migration, platelet aggregation, and assembly of ECM [32], [33]. From the above results, we speculate that the positive effect of βig-h3 is mediated through the up-regulation of integrin α2β1 activity.

βig-h3 contains four repetitive highly conserved FAS1 domains and a C-terminal arginyl-glycyl-aspartic acid (RGD) motif [14]. Previous studies reported that βig-h3 mediates cell functions through these motifs that interact with different integrins on various cell types. The second and the fourth FAS1 domains of βig-h3 mediate corneal epithelial cell adhesion by interacting with integrin α3β1. However, all four FAS1 domains of βig-h3 mediate fibroblastic cell adhesion by interacting with the integrin αvβ5 [15]–[17]. To gain insight into the molecular mechanisms, we assessed the effect of four FAS1 domain proteins on βig-h3-induced metastasis of osteosarcoma cells. The results showed that only the second FAS1 domain displayed comparable cell metastasis potential to the full length βig-h3 protein, indicating the existence of an integrin α2β1 -interacting motif in the second FAS1 domain of βig-h3 in osteosarcoma cells.

Integrins influence cell behavior not only by providing a docking site for ECM proteins at the cell surface, but also by acting to active signaling pathway regarding cell growth, survival and migration [34], [35]. To gain insight into integrin signaling mechanisms by which βig-h3 promotes metastasis potential of osteosarcoma cells, we investigated the activation of integrin downstream molecules. Various studies have suggested that integrins and their ligands collaborate closely with growth factors in transducing signals through the phosphoinositide 3-kinase (PI3K)-AKT pathway [36]–[38]. In our study, knockdown of βig-h3 caused reduction of AKT phosphorylation. At the same time, inhibition of PI3K resulted in inhibition of βig-h3-mediated AKT phosphorylation. These results indicated that βig-h3 may act via PI3K-dependent pathways to activate AKT. In addition, inhibition of PI3K resulted in inhibition of βig-h3-mediated cell adhesion, invasion and migration in osteosarcoma cells. These data highlight the involvement of PI3K/AKT signaling pathway as an intracellular pathway of βig-h3-mediated effects on metastasis of human osteosarcoma. Multiple intracellular pathways lead to PI3K/AKT signaling pathway activation. Ligand binding to integrins causes FAK phosphorylation, which in turn activates the PI3K/AKT signaling pathway and activation of PI3K/AKT signaling pathway by integrins has been described in other tumor cell types [39]–[41]. Our study presents that βig-h3 interacted with integrin α2β1. And blocking of integrin α2β1 with specific antibodies dismissed phosphorylating effect of βig-h3 on AKT. These results indicated that integrin α2β1 is involved in βig-h3 induced PI3K/AKT pathway activation in osteosarcoma cells. PI3K/AKT signaling is a promising therapeutic target for metastatic osteosarcoma [42], [43]. Therefore, this study revealed that downregulation of βig-h3 might contribute to the anti-metastatic therapy of human osteosarcoma through inhibiting PI3K/AKT signaling.

It is well recognized that the prognosis of patients with osteosarcoma are very poor due to the incurable nature of distant metastasis. Thus, preventing human osteosarcoma metastasis is an all-important issue nowadays. The results of this study identified that βig-h3 increases the adhesion, invasion and migration of human osteosarcoma cells via interacting with integrin α2β1 and activating PI3K/AKT signaling pathway. The discovery of βig-h3-mediated pathway helps us to understand the mechanism of human osteosarcoma metastasis and provides evidence for the possibility that βig-h3 can be a potential therapeutic target for osteosarcoma treatment.

## Materials and Methods

### Cell culture

The human osteosarcoma cell lines, Saos-2 and MG-63, were purchased from the American Type Culture Collection. Cells were cultured in RPMI 1640 medium (Gibco, Grand Island, NE, USA), supplemented with 10% FBS, 1% penicillin/streptomycin and 2%L-glutamine at 37°C in a humidified atmosphere of 5% CO_2_.

### Gene silencing

The sense sequence for βig-h3 small interfering RNAs (siRNAs) was 5′- CCUUUACGAGACCCUGGGATT-3′ and the anti-sense sequence was 5′-UCCCAGGGUCUCGUAAAGGTT-3′ (Ambion, Austin, TX, USA). βig-h3 siRNA was suspended in serum-free DMEM with LipofectAMINE 2000 reagent (Invitrogen, Carlsbad, CA, USA) for 20 min. The mixture was then aliquoted into 6-well plates containing pre-plated Saos-2 or MG63 cells to a final concentration of 40 nM of siRNA per well. Forty-eight hours later, cells were lysed for Western blot analysis. Silencer negative control siRNA (control siRNA) was used as a negative control under similar conditions (Ambion, Austin, TX, USA).

### Western blot analysis

Protein expression was analyzed by Western blotting as previously described [39]. In brief, cells were lysed in 1% n-octyl-p-D-glucopyrano-side (OG) buffer (20 mM Tris-HCl pH 8.0, 150 mM NaCl, 1% OG, 1 mM EDTA, 10 g/ml leupeptin, 2 g/ml aprotinin,1 mM PMSF). BCA Protein Assay Kit (Pierce Biotechnology, Rockford, IL, USA) was employed to determine the total protein density and equal amounts of proteins were separated by 10% SDS-polyacrylamide gel electrophoresis (SDS-PAGE), and then electransferred to polyvinylidene fluoride (PVDF) microporous membrane (Millipore, Boston, MA, USA). After blocking with 5% non-fat milk, the membrane was incubated for 2 h at room temperature with the designated antibody. Immunodetection was performed by using the Western-Light chemiluminescent detection system (Applied Biosystems, Foster, CA, USA).

### Real-time quantitative PCR

Total RNA was extracted from the cells with TRIzol reagents (Invitrogen, Carlsbad, CA, USA) and reverse transcribed into cDNA with a ReverTra Ace-a kit (Toyobo, Shanghai, China). All primers and probes were synthesized by Shanghai Sangon Co., and their sequences were as follows: βig-h3: forward primer, 5′-CATTGAGAACAGCTGCATCG-3′ and reverse primer, 5′-AGTCTGCTCCGTTCTCTTGG-3′; the first FAS1 domain (D-I),forward primer, 5′-AGGCCTGAGATGGAGGG-3′ and reverse primer, 5′-GTTGGTGATGGTGGAGA-3′; the second FAS1 domain (D-II), forward primer, 5′- TCCACCATCACCAACAAC-3′ and reverse primer, 5′-GATGAGCTACTCATC-3′; the third FAS1 domain (D-III), forward primer, 5′-GATGAGCTACTCATC-3′ and reverse primer, 5′-CATGACAGTCCCCAT-3′; and the fourth FAS1domain (D-IV), forward primer, 5′- CTGACCCCCCCAATG-3′ and reverse primer, 5′- GTTGGCTGGAGGCTG-3′; β-actin (control): forward primer, 5′-CCCAGCCATGTACGTTGCTA-3′ and reverse primer, 5′-TCACCGGAGTCCATCACGAT-3′. Real-time quantitative PCR was performed with SYBR green PCR reagent (Takara, Japan) and a Stratagene M×3005P Multiplex Quantitative PCR System (Agilent Technologies, Germany).

### Cell adhesion assay

The wells of a 96-well culture plate were coated with Matrigel (BD Biosciences, San Jose, CA, USA) at a concentration of 5 mg/ml and incubated at 4°C overnight. The coated wells were blocked with PBS containing 2% BSA for 30 min and then washed with PBS. Cells suspended in serum-free medium containing 0.1% BSA were added to the wells (2×10^4^/well) and incubated at 37°C, 5% CO_2_ for 30–60 min with or without antibodies. After removing medium and non-attached cells, 0.2% crystal violet was added for 10 min. The plate was gently washed with tap water and dried in air for 24 h. Then, 0.1 ml 5% SDS/50% ethanol was added for 20 min and then the plates were read at 540 nm.

### Cell invasion assay

The chemotactic cell invasion assay was performed using 24-well transwell units with an 8-µm pore size polycarbonate filter (Millipore, MA, USA). Each lower compartment of the transwell contained 600 ml of 0.5% FBS as chemo-attractant or 0.5% BSA as negative control in RPMI 1640. The upper side of a polycarbonate filter was coated with Matrigel (5 mg/ml in cold medium) to form a continuous thin layer. Prior to addition of the cell suspension, the dried layer of matrigel matrix was rehydrated with medium without FBS for 2 h at room temperature. Cells (1×10^5^) pre-incubated with antibodies were suspended in 0.1 ml RPMI 1640 containing 0.1% BSA and added into the upper compartment of the transwell unit and incubated for 24 h or 18 h at 37°C in a humidified atmosphere containing 5% CO_2_. Cells remaining in the upper compartment were completely removed with gentle swabbing. The number of cells that had invaded through the filter into the lower compartment was determined using a colorimetric crystal violet assay.

### Cell migration assay

Cells (2×10^6^) were plated in six-well plates and cultured to approximately 90% confluence. The cells were scraped with a pipette tip, washed several times in serum-free medium, and then examined under a phase contrast microscope (Olympus, Tokyo, Japan). The cells were re-fed with 10% FBS medium for 24 h or 18 h, and images were obtained.

### Immunofluorescence assay

Cells were allowed to attach for 8 h to glass coverslips. They were then fixed in 3.7% formaldehyde in PBS, permeabilized with 0.5% Triton X-100 and blocked with 1% BSA (Fraction V) in PBS for 1 h. Coverslips were incubated with the indicated antibodies at a 1∶500 dilution, or with rhodamine-phalloidin (Molecular Probes, Eugene, OR, USA) at a 1∶40 dilution in PBS for 20 min. Antibody-treated cells were washed in PBS and incubated with FITC or Texas red-conjugated secondary antibodies (Pierce Biotechnology, Rockford, IL, USA) at a 1∶500 dilution in PBS for 1 h. Cell nuclei were stained with DAPI (Vector Labs, Burlingame, CA). Finally, the cells were mounted using glycerol. Cells probed with rhodamine-phalloidin were washed and immediately mounted and observed by FV1000 laser scanning confocal microscopy (Olympus, Tokyo, Japan).

### Co-Immunoprecipitation (Co-IP) assay

The interactions between βig-h3 and integrin α2β1 in native cells were detected by ProFoundTM Mammalian Co-Immunoprecipi-tation Kit (Pierce Biotechnology, Rockford, IL, USA), according to the manufacturer's instructions. Briefly, Cells were lysed in 1% OG buffer. BCA Protein Assay Kit (Pierce Biotechnology, Rockford, IL, USA) was then used to determine the total protein density. Fraction of the lysates was saved as “input”. Then, aliquots of lysates (1 mL) were immunoprecipitated by 25 mL of protein A-agarose that was pre-bound with 2 mg of anti-βig-h3, anti-Integrin α2, anti-Integrinβ1, or anti-IgG antibodies, followed by four washes with the co-immunoprecipitation buffer. Immune complexes were eluted from coupling gel with Elution buffer and resolved by 10% SDS-PAGE. Integrin α2, Integrinβ1 or βig-h3 in the immune complexes was detected by Western blot using, respectively, anti-integrin α2 anti-integrin β1 or anti-βig-h3 antibodies (BD Biosciences, San Jose, CA, USA). anti-IgG were used as the negative control.

### Plasmid construction and transfection

Full-length βig-h3 (GenBank, NM_000358) and the four FAS1 domains were PCR-amplified, and the primers were designed as follows: Full-length βig-h3 (WT), forward primer, 5′- TTTTCTCGAGAGGCCTGAGATGGAGGG-3′(XhoI) and reverse primer, 5′- TAAATTCGAAATGATTVATCCTCTCTAA-3′(HindIII); the first FAS1 domain (D-I),forward primer, 5′-TTTTCTCGAG AGGCCTGAGATGGAGGG -3′(XhoI) and reverse primer, 5′-TAAATTCGAA GTTGGTGATGGTGGAGA -3′(HindIII); the second FAS1 domain (D-II), forward primer, 5′- TTTTCTCGAG TCCACCATCACCAACAAC -3′(XhoI) and reverse primer, 5′-TAAATTCGAA GATGAGCTACTCATC -3′(HindIII); the third FAS1 domain (D-III), forward primer, 5′-TTTTCTCGAG GATGAGCTACTCATC -3′(XhoI) and reverse primer, 5′-TAAATTCGAA CATGACAGTCCCCAT -3′(HindIII); and the fourth FAS1domain (D-IV), forward primer, 5′- 2TTTTCTCGAG CTGACCCCCCCAATG -3′(XhoI) and reverse primer, 5′- TAAATTCGAA GTTGGCTGGAGGCTG -3′(HindIII). The products of full-length βig-h3 (WT) and the four FAS1 domains were confirmed by sequencing (Shanghai Sangon, Shanghai, China) and then cloned into the pcDNA3.1 vector (Promega, Madison, WI, USA), respectively. After cells were grown to 60–80% confluency, transfection was performed using the LipofectAMINE 2000 reagent (Invitrogen, Carlsbad, CA, USA) according to the manufacturer's instructions. The pcDNA3.1 empty vector was used as a negative control under similar conditions.

### Cells stably expressing GFP

The cells were infected with a reconstructed pWPT–GFP–puro lentiviral vector, which contains a puromycin selection marker as previously reported [44]. For selection, 2 µg/ml puromycin was added to the medium, and after 14 days, the medium was changed to medium without puromycin. In this manner, the cells stably expressing GFP were established.

### Lung metastasis experiment

NOD/SCID immunodeficient mice were used for experimental lung metastasis experiment. Saos-2 human osteosarcoma cells expressing GFP were trypsinized and washed with PBS. Subsequently, 1×106 cells in 0.2 ml PBS were injected into the lateral tail vein. After 7 days, GFP fluorescence imaging was performed using the Xenogenin vivo Imaging System (IVIS 200, Xenogen, Alameda, CA, USA). GFP fluorescence images were analyzed with Igor image analysis software (Wavemetrics, Lake Oswego, OR, USA). The regions of interest were drawn over the signals, and the GFP fluorescence images were quantified in units of maximum photons per second per centimeter squared per steradian (p/s/cm2/s).

### Statistical analysis

Statistical analysis was performed using SPSS 13.0 statistical software. The results were expressed as mean values ± SD. And the Student's t-test or one-way ANOVA were used to evaluate the statistical significance in the groups. The differences were considered significant when *P*<0.05.

## References

[1] Messerschmitt PJ, Garcia RM, Abdul-Karim FW, Greenfield EM, Getty PJ (2009) Osteosarcoma. J Am Acad Orthop Surg 17 (8) 515–527. 19652033 10.5435/00124635-200908000-00005

[2] Kim HJ, Chalmers PN, Morris CD (2010) Pediatric osteogenic sarcoma. Curr Opin Pediatr 22 (1) 61–66. 19915470 10.1097/MOP.0b013e328334581f

[3] Harris MB, Gieser P, Goorin AM, Ayala A, Shochat SJ, et al (1998) Treatment of metastatic osteosarcoma at diagnosis: a Pediatric Oncology Group Study. J Clin Oncol 16: 3641–3648. 9817286 10.1200/JCO.1998.16.11.3641

[4] Meyers PS, Heller G, Healey G, Huvos A, Applewhite A, et al (1993) Osteogenic sarcoma with clinically detectable metastasis at initial presentation. J Clin Oncol 11: 449–453. 8445419 10.1200/JCO.1993.11.3.449

[5] Woodhouse EC, Chuaqui RF, Liotta LA (1997) General mechanisms of metastasis. Cancer 80 (Suppl. 8) 1529–1537. 9362419 10.1002/(sici)1097-0142(19971015)80:8+<1529::aid-cncr2>3.3.co;2-#

[6] Hanahan D, Weinberg RA (2000) The hallmarks of cancer. Cell 100: 57–70. 10647931 10.1016/s0092-8674(00)81683-9

[7] Chambers AF, Groom AC, MacDonald IC (2002) Dissemination and growth of cancer cells in metastatic sites. Nat Rev Cancer 2: 563–572. 12154349 10.1038/nrc865

[8] Bierie B, Moses HL (2006) Tumour microenvironment: TGF-β: The molecular Jekyll and Hyde of cancer. Nat Rev Cancer 6: 506–520. 16794634 10.1038/nrc1926

[9] Bernstein LR, Liotta LA (1994) Molecular mediators of interactions with extracellular matrix components in metastasis and angiogenesis. Curr Opin Oncol 6: 106–113. 7515692 10.1097/00001622-199401000-00015

[10] Ween MP, Lokman NA, Hoffmann P, Rodgers RJ, Ricciardelli C, et al (2011) Transforming growth factor-beta-induced protein secreted by peritoneal cells increases the metastatic potential of ovarian cancer cells. Int J Cancer 128 (7) 1570–84. 20521251 10.1002/ijc.25494

[11] Tang J, Wu YM, Zhao P, Jiang JL, Chen ZN (2009) βig-h3 interacts with α3β1 integrin to promote adhesion and migration of human hepatoma cells. Exp Biol Med 234: 35–39. 10.3181/0806-RM-187 18997105

[12] Shah JN, Shao G, Hei TK, Zhao Y (2008) Methylation screening of the TGFBI promoter in human lung and prostate cancer by methylation-specific PCR. BMC Cancer 8: 284. 18834524 10.1186/1471-2407-8-284 PMC2572632

[13] Skonier J, Bennett K, Rothwell V, Kosowski S, Plowman G, et al (1994) βig-h3, a transforming growth factor-β responsive gene encoding a secreted protein that inhibits cell attachment in vitro and suppresses the growth of CHO cells in nude mice. DNA Cell Biol 13: 571–84. 8024701 10.1089/dna.1994.13.571

[14] Kawamoto T, Noshiro M, Shen M, Nakamasu K, Hashimoto K, et al (1998) Structural and phylogenetic analysis of RGD-CAP/beta-ig-h3, a fasciclin like-adhesion protein expressed in chick chondrocytes. Biochim Biophys Acta 1395: 288–92. 9512662 10.1016/s0167-4781(97)00172-3

[15] Kim JE, Jeong HW, Nam JO, Lee BH, Choi JY, et al (2002) Identification of motifs in the fasciclin domains of the transforming growth factor-beta-induced matrix protein betaig-h3 that interact with the alphavbeta5 integrin. J Biol Chem 277: 46159–46165. 12270930 10.1074/jbc.M207055200

[16] Kim JE, Kim SJ, Lee BH, Park RW, Kim KS, et al (2000) Identification of motifs for cell adhesion within the repeated domains of transforming growth factor-beta-induced gene, betaig-h3,. J Biol Chem 275: 30907–30915. 10906123 10.1074/jbc.M002752200

[17] Park SW, Bae JS, Kim KS, Park SH, Lee BH, et al (2004) Beta igh3 promotes renal proximal tubular epithelial cell adhesion, migration and proliferation through the interaction with alpha3beta1 integrin. Exp Mol Med 36: 211–9. 15272232 10.1038/emm.2004.29

[18] Bae JS, Lee SH, Kim JE, Choi JY, Park RW, et al (2002) Betaigh3supports keratinocyte adhesion, migration, and proliferation through alpha3beta1 integrin. Biochem Biophys Res Commun 294: 940–8. 12074567 10.1016/S0006-291X(02)00576-4

[19] Ma C, Rong Y, Radiloff DR, Datto MB, Centeno B, et al (2008) Extracellular matrix protein βig-h3/TGFBI promotes metastasis of colon cancer by enhancing cell extravasation. Genes Dev 22: 308–321. 18245446 10.1101/gad.1632008 PMC2216691

[20] Ma J, Cui W, He SM, DuanYH, Heng LJ, et al (2012) Human U87 astrocytoma cell invasion induced by interaction of βig-h3 with integrinα5β1 involves calpain-2. Plos One 7 (5) e37297. 22629380 10.1371/journal.pone.0037297 PMC3357424

[21] Son HN, Nam JQ, Kim S, Kim IS (2013) Multiple FAS1 domains and the RGD motif of TGFBI act cooperatively to bind αvβ3 integrin, leading to anti-angiogenic and anti-tumor effects. Biochimica et Biophysica Acta 1833: 2378–2388. 23792174 10.1016/j.bbamcr.2013.06.012

[22] Zamilpa R, Rupaimoole R, Phelix CF, Somaraki-Cormier M, Haskins W, et al (2009) C-terminal fragment of transforming growth factor beta-induced protein (TGFBIp) is required for apoptosis in human osteosarcoma cells. Matrix Biology 28: 347–353. 19505574 10.1016/j.matbio.2009.05.004 PMC2756667

[23] Minn AJ, Gupta GP, Siegel PM, Bos PD, Shu W, et al (2005) Genes that mediate breast cancer metastasis to lung. Nature 436: 518–524. 16049480 10.1038/nature03799 PMC1283098

[24] Minn AJ, Kang Y, Serganova I, Gupta GP, Giri DD, et al (2005) Distinct organ-specific metastatic potential of individual breast cancer cells and primary tumors. J Clin Invest 115: 44–55. 15630443 10.1172/JCI22320 PMC539194

[25] Kimura H, Tome Y, Momiyama M, Hayashi K, Tsuchiya H, et al (2012) Imaging the inhibition by anti-β1 integrin antibody of lung seeding of single osteosarcoma cells in live mice. Int J Cancer 131 (9) 2027–33. 22323248 10.1002/ijc.27475

[26] Uehara F, Tome Y, Yano S, Miwa S, Mii S, et al (2013) A color-coded imaging model of the interaction of αv integrin-GFP expressed in osteosarcoma cells and RFP expressing blood vessels in Gelfoam vascularized in vivo. Anticancer Res 33 (4) 1361–6. 23564773

[27] Tome Y, Sugimoto N, Yano S, Momiyama M, Mii S, et al (2013) Real-time imaging of αv integrin molecular dynamics in osteosarcoma cells in vitro and in vivo. Anticancer Res 33 (8) 3021–5. 23898055

[28] Tome Y, Kimura H, Maehara H, Sugimoto N, Bouvet M, et al (2013) High lung-metastatic variant of human osteosarcoma cells, selected by passage of lung metastasis in nude mice, is associated with increased expression of α(v) β (3) integrin. Anticancer Res 33 (9) 3623–7. 24023288

[29] Nissinen L, Westermarck J, Koivisto L, Kähäri VM, Heino J (1998) Transcription of alpha2 integrin gene in osteosarcoma cells is enhanced by tumor promoters. Exp Cell Res 243 (1) 1–10. 9716443 10.1006/excr.1998.4128

[30] Scotlandi K, Serra M, Manara M, Nanni P, Nicoletti G, et al (1993) Human osteosarcoma cells, tumorigenic in nude-mice, express beta(1)-integrins and low-levels of alkaline-phosphatase activity. Int J Oncol 3 (5) 963–9. 21573460 10.3892/ijo.3.5.963

[31] Vihinen P, Riikonen T, Laine A, Heino J (1996) Integrin alpha 2 beta 1 in tumorigenic human osteosarcoma cell lines regulates cell adhesion, migration, and invasion by interaction with type I collagen. Cell Growth Differ 7 (4) 439–47. 9052985

[32] Tadokoro S, Shattil SJ, Eto K, Tai V, Liddington RC, et al (2003) Talin binding to integrin beta tails: A final common step in integrin activation. Science 302: 103–106. 14526080 10.1126/science.1086652

[33] Tanentzapf G, Brown NH (2006) An interaction between integrin and the talin FERM domain mediates integrin activation but no linkage to the cytoskeleton. Nat Cell Biol 8: 601–606. 16648844 10.1038/ncb1411

[34] Guo W, Giancotti FG (2004) Integrin signalling during tumour progression. Nat Rev Mol Cell Biol 5: 816–826. 15459662 10.1038/nrm1490

[35] Calderwood DA, Zent R, Grant R, Rees DJ, Hynes RO, et al (1999) The Talin head domain binds to integrin beta subunit cytoplasmic tails and regulates integrin activation. J Biol Chem 274: 28071–28074. 10497155 10.1074/jbc.274.40.28071

[36] Shaw LM (2001) Identification of insulin receptor substrate 1 (IRS-1) and IRS-2 as signaling intermediates in the alpha6beta4 integrin-dependent activation of phosphoinositide 3-OH kinase and promotion of invasion. Mol Cell Biol 21: 5082–5093. 11438664 10.1128/MCB.21.15.5082-5093.2001 PMC87234

[37] Izmailyan R, Hsao JC, Chung CS, Chen CH, Hsu PW, et al (2012) Integrin β1 mediates vaccinia virus entry through activation of PI3K/Akt signaling. J Virol 86 (12) 6677–87. 22496232 10.1128/JVI.06860-11 PMC3393588

[38] Krishnamurthy M, Li J, Fellows GF, Rosenberg L, Goodyer CG, et al (2011) Integrin {alpha}3, but not {beta}1, regulates islet cell survival and function via PI3K/Akt signaling pathways. Endocrinology 152 (2) 424–35. 21177833 10.1210/en.2010-0877

[39] Tang J, Wu YM, Zhao P, Yang XM, Jiang JL, et al (2008) Overexpression of HAb18G/CD147 promotes invasion and metastasis via α3β1 integrin mediated FAK-paxillin and FAK-PI3K-Ca^2+^ pathways. Cell Mol Life Sci 65: 2933–2942. 18695939 10.1007/s00018-008-8315-8 PMC7079791

[40] Murillo CA, Rychahou PG, Evers BM (2004) Inhibition of alpha5 integrin decreases PI3K activation and cell adhesion of human colon cancers. Surgery 136: 143–149. 15300173 10.1016/j.surg.2004.04.006

[41] Qin J, Tang J, Jiao L, Ji J, Chen WD, et al (2013) A diterpenoid compound, excisanin A, inhibits the invasive behavior of breast cancer cells by modulating the integrin β1/FAK/PI3K/AKT/β-catenin signaling. Life Sci 93 (18–19) 655–63. 24044886 10.1016/j.lfs.2013.09.002

[42] Jin S, Pang RP, Shen JN, Huang G, Wang J, et al (2007) Grifolin induces apoptosis via inhibition of PI3K/AKT signalling pathway in human osteosarcoma cells. Apoptosis 12 (7) 1317–26. 17333316 10.1007/s10495-007-0062-z

[43] Tsubaki M, Satou T, Itoh T, Imano M, Ogaki M, et al (2012) Reduction of metastasis, cell invasion, and adhesion in mouse osteosarcoma by YM529/ONO-5920-induced blockade of the Ras/MEK/ERK and Ras/PI3K/Akt pathway. Toxicol Appl Pharmacol 259 (3) 402–10. 22326785 10.1016/j.taap.2012.01.024

[44] Tang J, Guo YS, Zhang Y, Chen ZN, Jiang JL, et al (2012) CD147 induces UPR to inhibit apoptosis and chemosensitivity by increasing the transcription of Bip in hepatocellular carcinoma. Cell Death Differ 19 (11) 1779–90. 22595757 10.1038/cdd.2012.60 PMC3469060

